# Decomposition Dynamics of an Orangutan (*Pongo pygmaeus morio*) Carcass in a Tropical Forest: Implications for Conservation Practices

**DOI:** 10.1002/ece3.72662

**Published:** 2025-12-11

**Authors:** Sui P. Heon, Henry Bernard, Robert M. Ewers

**Affiliations:** ^1^ Institute of Tropical Biology and Conservation, Universiti Malaysia Sabah Kota Kinabalu Sabah Malaysia; ^2^ Rainforest Research Partnerships Sdn Bhd, Danum Valley Field Centre Lahad Datu Sabah Malaysia; ^3^ Georgina Mace Centre for the Living Planet Imperial College London Ascot UK

**Keywords:** Bornean orangutan, carcass decomposition, carrion ecology, primate conservation, tropical forests

## Abstract

Over the past decade, more than 600 rehabilitated Bornean Orangutans ( *Pongo pygmaeus morio* ) have been released into protected forests in Borneo. Releasing rehabilitant Bornean Orangutans into the wild is a standard conservation practice, yet monitoring postrelease survival remains a challenge. Limited data exist on post release survival, with many individuals classified as “missing but presumed dead” due to the absence of a carcass for confirmation. Detecting carcasses in tropical forests is particularly difficult due to dense vegetation and the narrow time frame for observing remains before complete decomposition or scavenger removal. Here, we report the first documented observation of a wild adult female Bornean Orangutan carcass decomposing process in the Danum Valley Conservation Area, Malaysian Borneo, on 21 May 2023. The approximately 30 kg carcass was monitored using camera traps and field observations. Decomposition was assessed using Payne's (1965) decomposition framework, the Total Body Score (TBS) system, and Accumulated Degree Days (ADD) to evaluate the influence of ambient temperature on decay. Decomposition progressed to the dry‐remains stage within 6 days, primarily driven by vertebrate scavengers such as the Asian water monitor lizard ( *Varanus salvator* ) and blow flies (*Calliphoridae*). This rapid decomposition rate challenges existing knowledge on the rate of decomposition of medium‐sized carcasses (> 10 kg) and suggests that the common practice of weekly monitoring for post‐release orangutans may be insufficient. Understanding decomposition processes and scavenger activity in tropical forests can improve carcass detection, refine mortality estimates for released Orangutans and other endangered species, and enhance conservation strategies for this critically endangered primate.

## Graphical abstract

We documented the complete decomposition process of a wild Bornean orangutan carcass in a lowland dipterocarp forest in Sabah, Malaysian Borneo. This represents the first detailed observation of great ape decomposition in Borneo. Our study provides a rare insight into the often‐overlooked process of carcass breakdown and decomposition in tropical rainforest ecosystems.

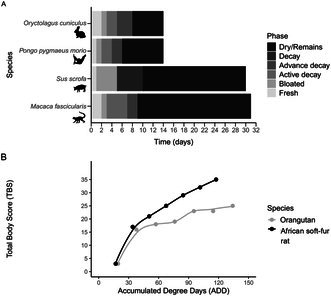

## 1 Introduction

Translocation and reintroduction are key conservation strategies for the critically endangered Bornean Orangutan ( *Pongo pygmaeus* ), widely implemented in Malaysia and Indonesia (Meijaard et al. 2012; Sherman et al. 2025). These great apes face severe threats, including habitat loss, illegal pet trade, hunting, and forest fragmentation (Meijaard et al. 2012). A common conservation practice involves rehabilitating displaced and confiscated Orangutans in rescue centers before releasing them into protected forests to support population recovery.

Best‐practice guidelines for wildlife rescue programs emphasize postrelease monitoring to assess survival and adaptation (IUCN 2013). However, tracking released orangutans remains challenging. Many individuals are fitted with implanted radio transmitters, yet detection rates are low, with 40%–95% never re‐encountered (Sherman et al. 2021). Released orangutans face major challenges, including diseases, exposure to pathogens, inadequate foraging abilities, negative social interactions, or predation, all of which may lead to significant mortalities (Russon 2008; Sherman et al. 2021). Reported survival estimates vary from 6% to 80%, but reliable long‐term records are lacking (Meijaard et al. 2012; Sherman et al. 2021). Some centers conduct intensive tracking for a few weeks before shifting to infrequent surveys, while others lack monitoring programs altogether (Sherman et al. 2020). The difficulty in confirming mortality further complicates survival estimates, as carcasses are rarely recovered. For example, in one of the rescue centers, 66% (25 of 38) of radio‐collared orangutans were unaccounted for 3 years postrelease and presumed dead, despite no carcass sightings (Sherman et al. 2020).

One potential explanation for the lack of carcass recoveries—beyond predation—is the rapid action of scavengers and decomposition in tropical forests. Globally, vertebrates remove up to 75% of carcasses within a short time (DeVault et al. 2003). In Borneo, most animals are likely to scavenge opportunistically, including mesocarnivores and raptors. However, previous studies have found that Malay civets ( *Viverra tangalunga* ), Bornean Bearded Pig ( *Sus barbatus* ), and Asian water monitors ( *Varanus salvator* ) are the most commonly detected vertebrate scavengers in this region (Galdikas 1978; Ellison 1986; Lim 2015; Twining et al. 2017), while Calliphoridae blowflies are the dominant invertebrate scavengers. Carcasses that escape vertebrate removal undergo six decomposition stages, from fresh to skeletal remains, driven by microbial and invertebrate activity (Payne 1965). This framework is widely used in carcass ecology and forensic studies, yet scavenging dynamics in tropical forests remain poorly documented.

Fewer than 20 Southeast Asian studies have examined carcass decomposition in tropical forests, with most focused on forensic applications (Azwandi et al. 2013; Heo et al. 2011; Zaini et al. 2025). Moreover, these studies have either excluded vertebrate scavengers or examined only large‐bodied scavengers while neglecting invertebrate activity (Ellison 1986; Lim 2015; Twining et al. 2017). Studies on vertebrate scavengers found that they detected and removed carcasses rapidly, usually within 48 h, for carcasses less than 1 kg. However, general scavenging patterns cannot be discerned, as some studies used baited experiments (Ellison 1986; Lim 2015), others used baited live traps (Twining et al. 2017), some were observational (Galdikas 1978), and carcass sizes varied considerably among studies. To our knowledge, no tropical study has simultaneously examined the roles of both vertebrate and invertebrate scavengers.

To date, there has only been one report on the decomposition of a wild Orangutan carcass by a forest scavenger, the Bearded pig ( *Sus barbatus* ; Galdikas 1978). Here, we document the complete decomposition process of a wild Orangutan carcass in a tropical forest. Although our observations are limited to a single individual, the opportunity to record such a process is rare—just two carcasses were observed in more than 8000 h of field observation over more than 5 years (Galdikas 1978)—and holds significant ecological value. Laboratory and field experiments on the decomposition of wild animal carcasses, particularly of critically endangered species, are neither ethical nor feasible. Hence, taking advantage of this unique event, our study addresses the following key questions: (1) Which scavenger species interacted with the carcass? and (2) What was the rate of decomposition of the Orangutan carcass? We then (3) compare that rate to other published and unpublished reports and data to assess the extent to which our single observation might be indicative of decomposition processes more generally. Understanding these processes can improve post‐release mortality assessments and enhance Orangutan conservation strategies, while also contributing new insights into carcass removal and scavenger dynamics in tropical ecosystems.

## 2 Methods

This study was conducted in primary lowland Dipterocarp Forest at Danum Valley Conservation Area (4.96474 N, 117.80385 E; 150–1000 m a.s.l), Sabah, Malaysian Borneo, in May 2023. The vegetation is dominated by trees of the Dipterocarpaceae family with Euphorbiaceae abundant in the shrub layer (Newberry et al. 1992). The climate in Danum Valley is aseasonal, with a relatively uniform distribution of precipitation of approximately 2700 mm.year^−1^ (Walsh and Newberry 1999). On May 21, a known adult female Bornean Orangutan ( *Pongo pygmaeus* ), approximately 30 kg in weight and 1.2 m in body length (Morton et al. 2013), was found dead ~120 m from the field center after distress calls from her infant were heard. The incident was reported to the park authorities who initiated a search and rescue for the infant. The cause of death was unknown but presumed to be illness or senescence, as she had been observed spending unusual time on the forest floor prior to death (J. Anson, pers. comm.). She was last seen alive on May 20 with no visible injuries. The site was closed to visitors within 24 h due to strong putrefaction odor.

A Reconyx HC500 motion‐triggered camera (Reconyx, USA) was deployed at the carcass about 1 m from the carcass, set to five‐image bursts every 15 min and upon motion. Independent scavenger visits were defined as detections > 30 min apart. On Day 4, the camera was repositioned ~2 m after an Asian water monitor ( *Varanus salvator* ) moved the carcass on the evening of Day 3. As a result, we may have missed some detections in that time period. A phone camera (Samsung, South Korea) supplemented monitoring in later stages when the limbs detached. The observer conducted daily visits to assess carcass condition and observe invertebrate scavenger activity, using randomized varying times to reduce disturbance.

Camera trap images were used to validate the classification of decomposition stages (Chen et al. 2025). We assessed decomposition using two standard methods. First, we qualitatively recorded decomposition stages following Payne's (1965) framework, as weighing the carcass was not feasible. These stages were then compared to published decomposition studies (Ahmad et al. 2011; Rimbin et al. 2020; Silahuddin et al. 2015) conducted in other lowland Dipterocarp forests in Malaysia and Indonesia under similar conditions (i.e., no scavenger exclusion and no manipulations of carcass condition). From these studies, we extracted information on the carrion decomposition phases and their duration. Although the carcass types and sizes varied among these studies, the reported stages of decomposition are more generalizable and provide a useful basis for comparing overall patterns.

Secondly, we applied the Total Body Score (TBS) system and Accumulated Degree Days (ADD) method (see Megyesi et al. 2005) to quantify decomposition relative to temperature. The TBS method was selected because it allows for a standardized quantification of carcass decay progression while accounting for variation in temperature (Dawson et al. 2002). TBS was calculated daily based on the head/neck, trunk, and limbs. After scavenger removal of the limbs, limb scores (10 points) were excluded, reducing the maximum TBS from 35 to 25. This loss in mass may lead to a slight underestimation of the overall TBS scoring (as discussed in Dawson et al. 2002). All TBS assessments were conducted by the first author, with scoring supported by camera trap images to reduce potential observer bias (see Dabbs et al. 2016).

We obtained temperature data from the Danum Weather Station (O'Brien et al. 2025) located approximately 120 m from the carcass site. The weather station recorded daily maximum and minimum temperatures, which were then used to calculate accumulated degree days (ADD). The carcass was situated under intact canopy, and previous studies indicate that forest interior temperatures deviate only slightly from ambient conditions (Ismaeel et al. 2023). We then used these data to examine whether the rate of decomposition was comparable to that observed for 15 g small mammal in the same forest (Heon et al. 2025). We calculated Accumulated Degree Days (ADD) for each day of decomposition as follows:

$$\mathrm{ADD}=\sum \left(T_{\mathrm{avg}}-T_{\text{base}}\right),$$

where.

$$T_{\mathrm{avg}}=\left(T_{\max}+T_{\min}\right)/2.$$

We set *T*_base_ at 9.5°C, the minimum developmental threshold for *Chrysomya rufifacies* (Diptera: Calliphoridae), the dominant dipteran scavenger in Malaysian rainforests (Ahmad et al. 2011; Yanmanee et al. 2016). *T*_max_ is the maximum temperature of the day while *T*_min_ was the minimum temperature of the same day. For decomposition studies, ADD is used instead of daily temperature measurements because decomposition is driven by the cumulative thermal energy over time within the carcass, as opposed to daily mean or extreme temperature.

## 3 Results

**FIGURE 1:**
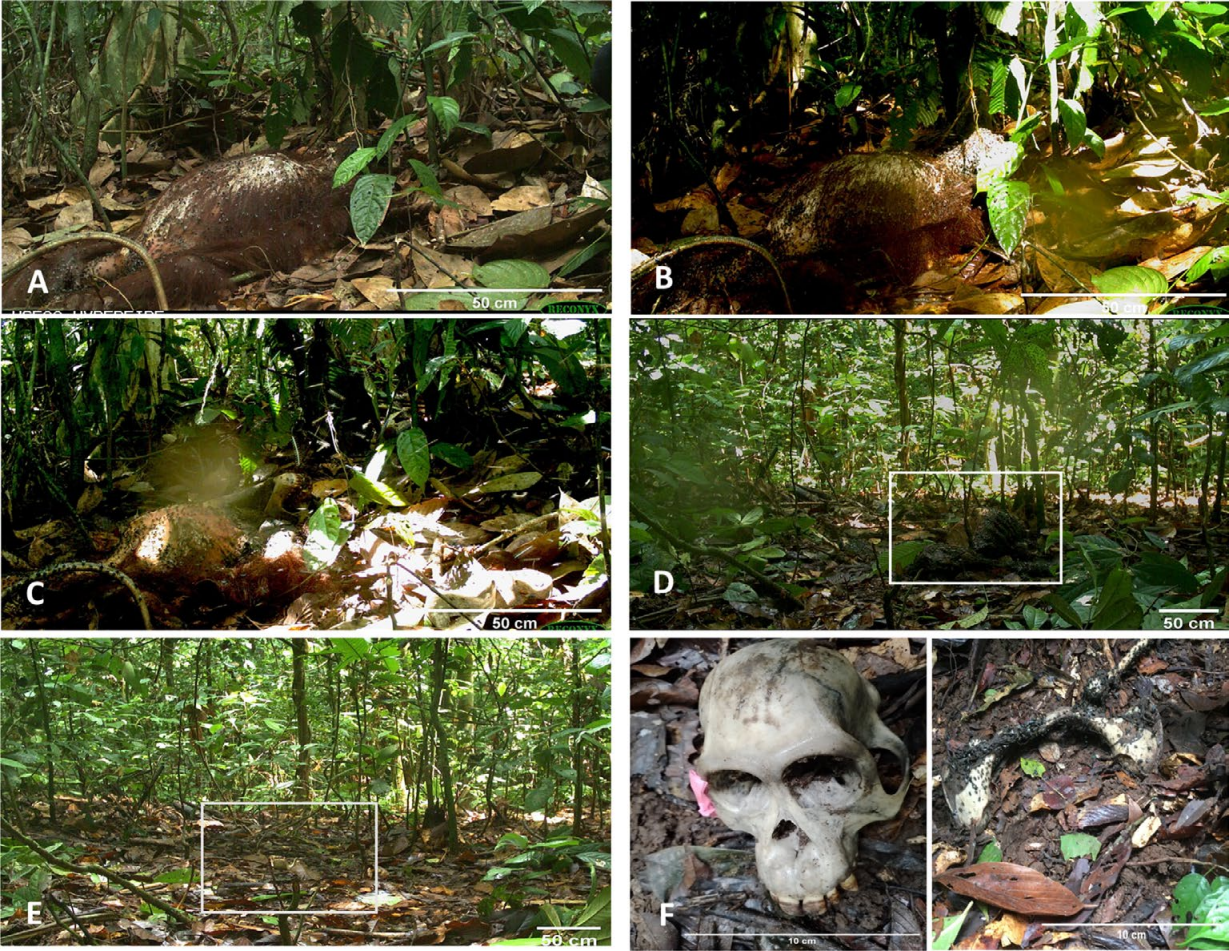
Decomposition stages of a wild Orangutan carcass: (A) Fresh stage: Day 0–1; (B) Bloat: Day 1–2; (C) Active decay with skull emerging: Day 2–4; (D) Advanced decay:Day 4; (E) Dry—limbs removed and skeletal elements visible (inset): Day 6; (F) Skeletal remains: Day 10 onwards.

**FIGURE 2:**
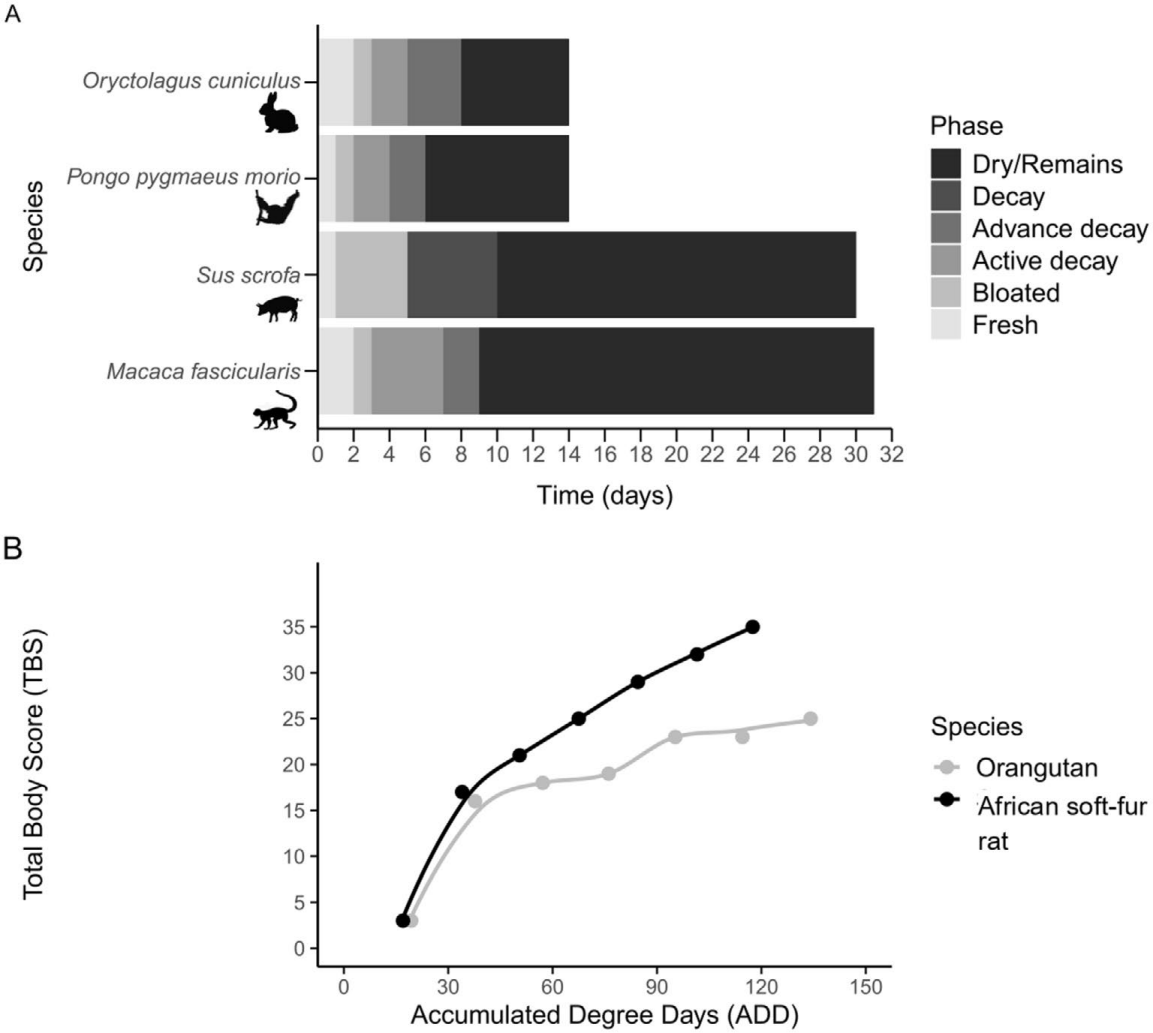
(A) Decomposition timeline of a wild Bornean orangutan carcass following the classification of Payne (1965) compared to selected studies on other taxa in Southeast Asia: Rabbit ( *Oryctolagus cuniculus* ) (2 kg; Ahmad et al. 2011), domestic pig ( *Sus scrofa* ) (35 kg; Rimbin et al. 2020), and long‐tailed macaque ( *Macaca fascicularis* ) (5.7 kg; Silahuddin et al. 2015). For the domestic pig, the active and advanced decay phases were combined as “Decay” in the source study. These comparisons illustrate general decomposition patterns among taxa, but we note that differences in sampling sites and methods restrict the extent to which direct comparisons can be made. (B) Decomposition pattern of the orangutan carcass and an African soft‐fur rat ( *Mastomys natalensis* ), a small mammal carcass (< 1 kg) using the Total Body Score (TBS) method and Accumulated Degree Days (°C days) over time. The maximum TBS for the orangutan was 25 and for the small mammal was 35.

The Orangutan carcass progressed through all stages of decomposition—from fresh to dry—in just 6 days (Figure 1). This decomposition rate appears faster than reported for some other taxa under different conditions in the region, despite the Orangutan's relatively larger body size (Figure 2A). The carcass transitioned through the bloat (Day 1–2) and active decay stages (Day 2–3) at a faster rate than in other studies; these stages lasted longer. The first scavengers to arrive were blowflies (*Chrysomya rufifacies* and *Chrysomya megacephala*), which were dominant from Day 1 to Day 3, corresponding to the fresh and active decay stages. By Day 3, dipteran larvae had consumed much of the soft tissue, exposing the Orangutan's skull. Time‐lapse footage revealed that bloat occurred twice during the early decomposition phase—specifically in the late evening and early morning—likely due to gas accumulation and subsequent rupture (Figure 1).

Camera traps recorded three vertebrate scavenger species. The Asian water monitor ( *Varanus salvator* ) appeared seven times, repeatedly visiting the carcass during the active and advanced decay stages. While we were unable to quantify the biomass removed by each scavenger—since weighing the carcass in situ would have disrupted invertebrate activity—camera observations showed two water monitors dismembering and carrying off the orangutan's limbs. The remaining trunk and head region decomposed in place, primarily via dipteran larval activity. Predatory staphylinid beetles (*Staphylinidae*) were observed feeding on the fly larvae on Day 4 when the carcass was at the advanced decay stage.

A Malay civet ( *Viverra tangalunga* ) was also observed on Day 2, engaging in scent‐rubbing behavior—likely a form of territorial or social communication—but it did not return in subsequent days. By Day 4, fly pupae were visible on the Orangutan's hair and carcass. On Day 5, some pupae had dropped to the surrounding soil, and by Day 6, the carcass had been reduced to bone fragments, with no further visible scavenger or invertebrate activity.

Two years after decomposition, the bones were distributed across an area of roughly 10 m^2^ and remained largely undisturbed. The skull was still visible, while other bone fragments were partially buried under dense leaf litter. There were no signs of bone gnawing, but algal growth and tiny cavities, presumably created by termites, suggest postdecomposition decay processes were underway.

Our assessment of the relationship between time and decomposition phases (Figure 2B) showed that both the Orangutan and small mammal carcass reached their maximum Total Body Score (TBS)—25 for the Orangutan and 35 for the small mammal—within a range of 100–140 accumulated degree days (ADD). The most rapid decomposition occurred within the first 100°C days, after which the rate notably decreased.

## 4 Discussion

The rapid decomposition of the Orangutan carcass in our study was likely due to the combined scavenging activity of both invertebrates and vertebrates. Dismemberment by Asian water monitors increased carcass accessibility, thereby accelerating decomposition by invertebrates. In comparison, carcasses in previous studies decomposed more slowly, likely because dipteran larval development requires 2–3 days before reaching optimal feeding levels (Ivorra et al. 2023).

The time‐lapse camera trap function also recorded two distinct episodes of bloating. This is a rarely documented phenomenon and was likely caused by a sequence of events: gas accumulation from high microbial activity during the day, a reduction in activity after nightfall, and a subsequent surge the following morning, which ultimately led to carcass tissue rupture and the release of putrescent gases. The first vertebrate scavengers were only detected after the bloat stage and may have been attracted at this point from the release of putrescent gases during carcass deflation (Kane et al. 2014).

Although there are no obligate scavengers in Bornean tropical forests, our findings support the role of the Asian water monitor ( *Varanus salvator* ) as a likely important scavenger of orangutan carcasses. This observation aligns with previous reports by Lim (2015) and Twining et al. (2017), who found this mesopredator frequently scavenging on small mammal and bird carcasses in Borneo. As generalist feeders with a highly developed sense of smell, Asian water monitors are adept at detecting carcass (Cairncross et al. 2024). The scent of putrescence, strongest after the bloat stage when invertebrate activity has ruptured the carcass, likely facilitates this detection (Kane and Kendall 2017).

Interestingly, the monitors did not consume the entire carcass, possibly due to the carcass size, which is nearly double the body weight of an adult monitor. This is consistent with the observation by Fitzsimons and Thomas (2016) that a monitor lizard made repeated visits to remove small amounts of tissue from a wild pig carcass (> 50 kg) in Eastern Sabah. Removed limbs were not consumed on site, possibly due to intraspecific competition. Contrary to expectations, no other vertebrate scavengers were detected, despite the carcass representing a nutrient‐rich resource. Notably, this study took place shortly after an African Swine Fever outbreak in Sabah, which led to a 90% decline in the wild pig population (Daniel et al. 2025; Ewers et al. 2021). The bearded pig ( *Sus barbatus* ), a crepuscular species that is widespread and found in high densities in forest habitats, and a presumed scavenger, may have competed for the carcass under normal conditions, as observed by Galdikas (1978). Bearded pigs possess strong olfactory senses and are known for their broad diets, which include carcasses (Kurz and Goosens 2002). However, their absence from the landscape post‐outbreak leaves its current role as a scavenger of large carcasses uncertain.

We observed Staphylinid beetles predating the larvae of flies on the carcass, illustrating how energy and nutrients from carcasses are transferred up the food chain via a brown web. Staphylinids are commonly associated with large larval masses on carcasses and have previously been observed predating on dipteran eggs and larvae at the early and advanced stages of decomposition (Heo et al. 2011; Madinah and Rahim 2018; Von homermann et al. 2021). However, the potential role that predatory invertebrates like Staphylinids might play in controlling the rate of carrion decomposition remains unexplored.

Temperature is an important driver of decomposition. The consistently warm temperatures (25°C–29°C) in Danum's forests likely supported high invertebrate activity, rapid dipteran larval development, and microbial decomposition. *Chrysomya rufifacies* is known to thrive between 16°C–34°C, with oviposition occurring within 0.5 days and a full life cycle completed in approximately 8 days (Zhang et al. 2017). Consistent with our findings, Ivorra et al. (2023) reported that *Chrysomya* larvae require 61.9°C–132°C degree days to complete the larval feeding stage. Beyond 132°C degree days, larvae transition to the pupal stage and burrow into the surrounding soil.

Visual comparison of our results to other published records shows that decomposition rates and scavenger outcomes may differ even under similar climatic and forest conditions. This variability likely reflects the limited number of studies conducted in natural environments, where carcass detection is inherently challenging (Turner et al. 2017); even long‐term field studies have struggled to monitor mortality in the wild due to the difficulty of detecting freshly deceased animals (Galdikas 1978; Huang et al. 2014; Milton 1990). Experimentally placing carcass proxies is a practical means of studying mortality and scavenging dynamics. However, most published studies have excluded either vertebrates or invertebrates from the decomposition process, leaving a gap in our understanding of natural carcass removal when both classes of scavenger openly compete. Alternatively, integrating theoretical and empirical approaches to advance our understanding of scavenger dynamics could be explored (Kane and Kendall 2017).

Overall, our study suggests that the rapid decomposition of carcasses may partly explain the lack of confirmed mortality cases among rehabilitated orangutans. This highlights the importance of reassessing the frequency and methods of monitoring released individuals. Intensive post‐release monitoring programs, such as those successfully applied on the Western Lowland Gorillas (Gorilla gorilla gorilla), which involve daily monitoring of released individuals for up to 3 years post‐release, may significantly improve estimates of long‐term survivorship (King et al. 2014). However, such approaches may not be cost‐effective or logistically feasible in the dense tropical forests of Borneo. Alternative methods which are currently being employed include the use of transponder‐telemetry equipment, targeted searches for the released individuals, and improved disease monitoring at rescue centers. Regardless of the method that might be most locally appropriate, our findings emphasize the need for more reliable and intensive monitoring systems to better understand postrelease outcomes. While rehabilitation can be a vital conservation tool, knowing whether it is a success depends heavily on the ability to detect mortality and survival events in the wild.

Using weekly monitoring to ascertain the fate of released Orangutans may be too infrequent to adequately detect mortality events. We found almost all evidence of a mortality event had disappeared within just 6 days, with even the smell of the carcass having dissipated and the only remains being inconspicuous bones on the forest floor. We do, of course, acknowledge that our study is an opportunistic one and based on a single carcass, which restricts generalizing our findings regarding Orangutan decomposition, scavenger behavior, and removal rates in tropical forests. However, the patterns in our data are reasonably consistent with observations from other reports and data on both large and small mammal carcass decomposition trends and provide some assurance that what we observed was not an aberrant event. Importantly, this study is among the first to document the decomposition of an Orangutan in the wild, and our results highlight the need to re‐evaluate current monitoring practices for rehabilitated Orangutans.

## Author Contributions

**Sui P. Heon:** conceptualization (lead), data curation (lead), formal analysis (lead), funding acquisition (lead), investigation (lead), methodology (lead), project administration (lead), visualization (lead), writing – original draft (lead), writing – review and editing (lead). **Henry Bernard:** conceptualization (supporting), supervision (equal), validation (equal), writing – review and editing (equal). **Robert M. Ewers:** conceptualization (equal), funding acquisition (equal), methodology (equal), resources (equal), supervision (equal), validation (equal), visualization (equal), writing – review and editing (equal).

## Funding

This work was supported by the Association for Tropical Biology and Conservation, ATBC SEED GRANT.

## Conflicts of Interest

The authors declare no conflicts of interest.

## Data Availability

The data used in this study are available in Zenodo at http://doi.org/10.5281/zenodo.16250654.
